# Robotic esophageal diverticulectomy with Heller myotomy and Dor fundoplication for giant epiphrenic diverticulum: a case report

**DOI:** 10.1093/jscr/rjag531

**Published:** 2026-06-29

**Authors:** Ashraf Maghrabi

**Affiliations:** Department of Surgery, Faculty of Medicine, King Abdulaziz University, PO Box 80215, Jeddah 21589, Saudi Arabia

**Keywords:** epiphrenic diverticulum, robotic surgery, Heller myotomy, Dor fundoplication, esophageal motility disorder

## Abstract

Epiphrenic esophageal diverticula are rare pulsion diverticula often associated with esophageal motility disorders. We report a 71-year-old man with a 7-year history of reflux, dysphagia, nocturnal regurgitation, and 12-kg weight loss over 8 months. Imaging revealed a 5 × 7 cm distal esophageal diverticulum, a 5-cm hiatal hernia, and findings suggestive of an esophageal motility disorder with a hypertensive lower esophageal sphincter. The patient underwent robotic diverticulectomy, Heller myotomy, hiatal hernia repair, and Dor fundoplication. Postoperative contrast study showed no leak, and he was discharged on Day 4. At 6-month follow-up, he remained asymptomatic with improved quality of life; the Gastroesophageal Reflux Disease - Health-Related Quality of Life (GERD-HRQL) score improved from 42 preoperatively to 18 at 30 days postoperatively. This case adds to the limited literature on robotic management of giant epiphrenic diverticula, illustrating that the robotic approach offers superior mediastinal visualization and that combined diverticulectomy with myotomy and fundoplication effectively addresses both anatomical and functional pathology.

## Introduction

Epiphrenic diverticula are pulsion outpouchings of the distal 10 cm of the esophagus, involving herniation of mucosa and submucosa through the muscular wall. Their prevalence is under 0.5% in the general population [1, 2]. Most cases are associated with esophageal motility disorders, such as achalasia, diffuse esophageal spasm, or hypertensive lower esophageal sphincter (LES), present in 75%–100% of patients [3]. The underlying mechanism is increased intraluminal pressure from functional obstruction at the gastroesophageal junction, which promotes progressive herniation at points of anatomical weakness [4]. Giant epiphrenic diverticula (>5 cm) are rare but may cause severe symptoms, including dysphagia, regurgitation, aspiration pneumonia, and marked weight loss [4]. Surgical treatment must address both the diverticulum and the underlying motility disorder, typically via myotomy, combined with an anti-reflux procedure to prevent postoperative gastroesophageal reflux [5, 6]. Minimally invasive approaches—especially robotic surgery—provide enhanced visualization and instrument articulation, which are critical for precise dissection in the confined high mediastinum [7, 8]. Here, we report a case of successful robotic management of a giant epiphrenic diverticulum, with the patient remaining symptom-free at 6 months follow-up.

## Case report

A 71-year-old man presented with a 7-year history of progressive reflux, including heartburn, nocturnal regurgitation exacerbated when supine, moderate dysphagia to solids, recurrent choking episodes, and 12 kg of unintentional weight loss over 8 months. His medical history was notable for well-controlled hypertension, type 2 diabetes mellitus, hypothyroidism, and prior laparoscopic cholecystectomy. On examination, he appeared thin, with signs of malnutrition including temporal wasting and reduced subcutaneous fat. Laboratory evaluation revealed hypoalbuminemia (albumin 3.0 g/dl), with otherwise normal complete blood count and metabolic panel.

**Figure 1:**
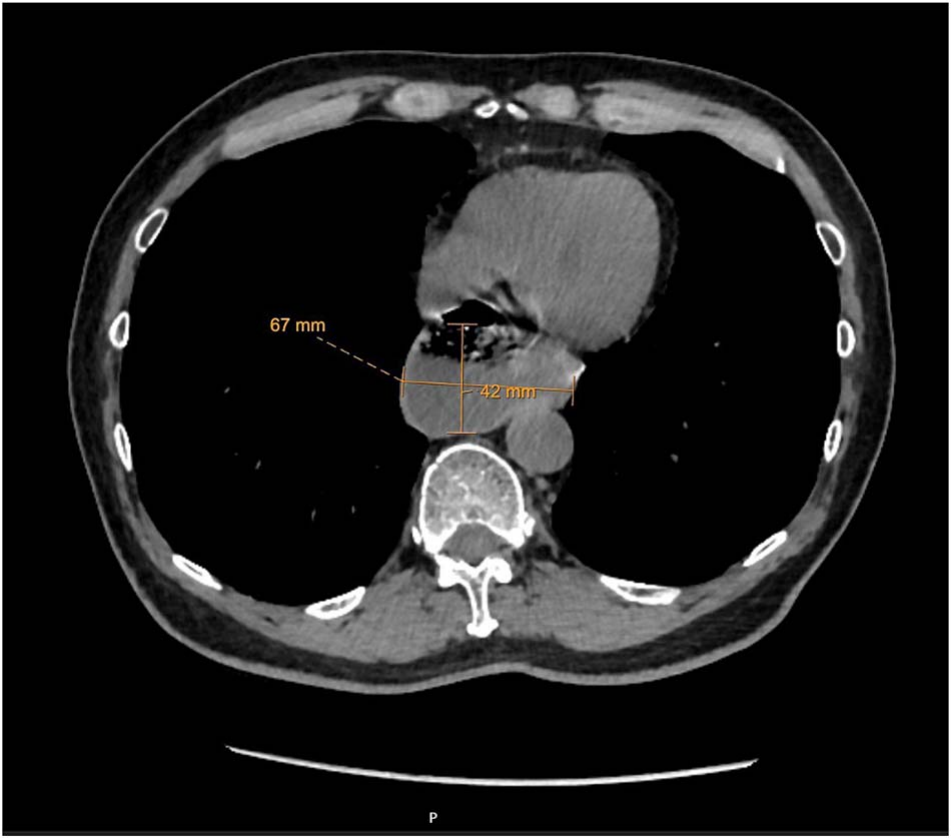
Axial CT demonstrating a 5 × 7 cm distal esophageal diverticulum with a 5-cm hiatal hernia.

Barium swallow showed a dilated, tortuous esophagus with a large distal diverticulum located 33–37 cm from the incisors, a 5-cm hiatal hernia, and grade III gastroesophageal reflux on the radiologic reflux grading system [9]. Computed tomography (CT) of the chest and abdomen confirmed a 5 × 7 cm air- and fluid-filled diverticulum arising from the distal esophagus, alongside the hiatal hernia (Fig. 1). Upper gastrointestinal endoscopy revealed retained food debris, a hypertensive LES with moderate resistance to scope passage, and a large diverticulum with a wide ostium. Esophageal high-resolution manometry, which is the standard preoperative investigation to confirm an associated motility disorder and guide the extent of myotomy, was attempted twice but could not be completed because of patient intolerance. The underlying motility disorder was therefore presumed on the basis of the clinical presentation, the dilated tortuous esophagus, and the endoscopic finding of a hypertensive LES with resistance to scope passage. The preoperative GERD-HRQL score was 42 [10].

**Figure 2:**
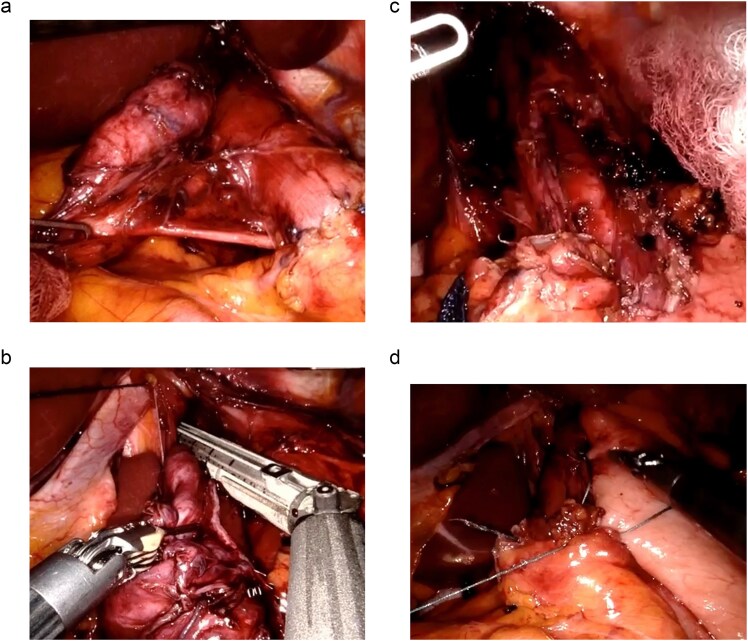
Intraoperative images. (a) Large epiphrenic diverticulum after mediastinal dissection. (b) Diverticular neck transected with a purple 60-mm Tri-stapler. (c) Completed 8-cm esophagogastric myotomy. (d) Completed anterior 180° Dor fundoplication.

After nutritional optimization, the patient underwent robotic esophageal diverticulectomy, Heller myotomy, posterior hiatal hernia repair, and anterior Dor fundoplication using the da Vinci Xi system (Supplementary Video S1). He was positioned in split-leg low lithotomy with reverse Trendelenburg. Four 8-mm robotic ports and a 12-mm assistant port were placed. Liver retraction was achieved using a crural stitch technique, with a suture passed through the right crus and left lobe of the liver, then secured to the abdominal wall. Circumferential dissection of the hiatus was performed, identifying both crura, and a 5-cm hiatal hernia was reduced. The phrenoesophageal membrane was incised, and dissection continued into the posterior mediastinum. The diverticular sac was mobilized circumferentially, taking care to preserve the vagal trunks and avoid injury to the esophageal wall (Fig. 2a). Intraoperative endoscopy was used to confirm complete resection of the diverticulum and to prevent esophageal narrowing during stapling. The diverticular neck was transected using a purple 60-mm Tri-stapler perpendicular to the esophageal axis (Fig. 2b). The staple line was reinforced with running 2–0 V-Loc suture in a Lembert fashion. An 8-cm esophagogastric myotomy was performed with endoscopic guidance, extending 6 cm onto the esophagus and 2 cm onto the gastric cardia (Fig. 2c). Posterior crural repair was completed with interrupted 0 Ethibond sutures, followed by an anterior 180° Dor fundoplication, secured to both crura and the myotomy edges (Fig. 2d).

Postoperatively, the patient received intravenous proton pump inhibitors and was monitored in the surgical ward. Clear fluids were initiated on Day 1. A water-soluble contrast study on Day 2 showed no extravasation, with free passage of contrast into the small bowel. The diet was advanced to full liquids on Day 3. He was discharged on Day 4 tolerating full liquids, with prescriptions for oral proton pump inhibitors and a prokinetic agent. At 6-month follow-up, he remained completely symptom-free, with resolution of dysphagia and regurgitation, had regained 8 kg, and reported excellent quality of life on a regular, unrestricted diet. The GERD-HRQL score improved from 42 preoperatively to 18 at 30 days postoperatively, consistent with the symptomatic improvement and with a good response to low-dose (20 mg) proton pump inhibitor; quality of life was not formally reassessed at the 6-month visit.

## Discussion

This case highlights successful robotic management of a giant epiphrenic diverticulum with excellent 6-month outcomes. Epiphrenic diverticula arise from increased intraluminal pressure due to underlying motility disorders, making myotomy essential to address the pathophysiology and prevent recurrence [3, 5]. Recurrence rates of up to 20% have been reported when myotomy is omitted, emphasizing its critical role [5]. A partial fundoplication provides dual benefits: preventing postoperative gastroesophageal reflux after myotomy and reinforcing the diverticulectomy staple line [6, 11].

Minimally invasive approaches are now the preferred strategy for managing epiphrenic diverticula. Rosati *et al.* first reported laparoscopic transhiatal diverticulectomy with myotomy and fundoplication in 1998, demonstrating its feasibility and safety [6]. Robotic platforms offer additional advantages, including high-definition three-dimensional visualization, seven degrees of instrument articulation, and tremor filtration—features that facilitate precise mediastinal dissection and stapler placement in confined anatomical spaces [7, 8].

Recent reports support the safety and efficacy of robotic management for giant epiphrenic diverticula. Abughararah *et al.* described a 57-year-old man with a large thoracic esophageal diverticulum treated with robotic diverticulectomy and myotomy, achieving complete symptom resolution at 11 months [12]. Farooqi *et al.* reported a 68-year-old woman with a 7.5 × 6 cm diverticulum complicated by severe pericardial adhesions, successfully managed robotically and discharged on postoperative Day 5 [13]. Singh *et al.* described total robotic transhiatal excision of an 8 × 6 × 7 cm left-sided diverticulum, with oral intake initiated on Day 1, demonstrating rapid recovery [14]. Our case further supports these findings, demonstrating excellent short-term outcomes with the robotic approach.

The Dor fundoplication was chosen because it provides effective anti-reflux protection while covering the anterior esophageal staple line and myotomy site, thereby reducing the risk of staple line leak [11].

This report has several limitations. It describes a single patient, which limits generalizability. Esophageal manometry could not be completed because of patient intolerance, so the underlying motility disorder was presumed on clinical, endoscopic, and radiologic grounds rather than confirmed manometrically. Follow-up was relatively short at 6 months, and quality of life (GERD-HRQL) was reassessed only at 30 days rather than at later time points; longer follow-up with objective physiologic testing would strengthen the assessment of durability.

In conclusion, this case adds to the limited but growing literature on robotic management of giant epiphrenic diverticula, supporting the safety and feasibility of the approach and its technical advantages for precise mediastinal dissection, while higher-level comparative evidence is still needed.

## Supplementary Material

JSCR_rjag531 Video 1 Robotic esophageal diverticulectomy with Heller myotomy and Dor fundoplication.
